# Carbonic anhydrase 12 gene silencing reverses the sensitivity of paclitaxel in drug-resistant breast cancer cells

**DOI:** 10.1080/21655979.2021.1995575

**Published:** 2021-12-02

**Authors:** Ting Huang, Lijuan Tang, Huan Wang, Lu Lin, Jing Fu

**Affiliations:** a Department of Breast Surgery, Sichuan Academy of Medical Sciences & Sichuan Provincial People’s Hospital; School of Medicine, University of Electronic Science & Technology of China; b Department of General Surgery, SiChuan TianFu New District People’s Hospital

**Keywords:** Carbonic anhydrase 12, siRNA, breast cancer, paclitaxel resistant, apoptosis

## Abstract

This study aimed to investigate the effects of carbonic anhydrase 12 (CA12)-siRNA on the paclitaxel sensitivity of breast cancer cells. Normal mammary glandular cell (MCF-10), breast cancer cell (MCF-7), and paclitaxel-resistant breast cancer cells (MCF-7 TaxR) were cultured in experimental control group. Western blot was adopted to detect the expressions of CA12 protein and apoptosis-related proteins in mitochondrial pathway of MCF-10, MCF-7, and MCF-7 TaxR cells. The methylthialazole tetrazolium (MTT) method was used to measure cell proliferation. The apoptosis of MCF-7 and MCF-7 TaxR cells was observed in phase contrast microscope, fluorescence inverted phase contrast microscope, and flow cytometry (FACS). The results showed that CA12 protein expression in MCF-7 and MCF-7 TaxR cells was significantly higher than that in MCF-10 cell. The growth rate of CA12-siRNA treated MCF-7 TaxR cells with paclitaxel (PTX) co-culture was markedly declined at 48 hours. Phase contrast microscope, fluorescence inverted phase contrast microscope, and FACS showed that apoptotic cells in the CA12-siRNA treated MCF-7 TaxR groups were significantly higher than that in CA12-siRNA treated MCF-7 cells. The expressions of pro-apoptotic proteins, Bax and Bid, were dramatically increased in CA12 siRNA treated MCF-7 TaxR cells. The expression quantity of the downstream effective molecules caspase-9, caspase-7, and the activated proteins of poly (ADP-ribose) polymerase (PARP), also were significantly increased. Our results indicated that the application of PTX combined silencing CA12 was able to activate the mitochondrial apoptosis pathway and promote MCF-7 TaxR apoptosis. CA12 silencing in the PTX-resistant breast cancer cell can reverse the sensitivity of PTX.

## Introduction

Breast cancer is a cancer with the highest incidence in most countries of the world, accounting for a quarter of all cancers in women [1]. The morbidity and mortality of breast cancer in China are continue to rise, and total new and death cases of breast cancer account for about one-tenth of the world every year [2]. Chemotherapy is one of the main methods for comprehensive treatment of breast cancer. However, multidrug resistance (MDR) mediated by various mechanisms of tumors is the main obstacle for treatment failure in most patients [3].

Main mechanism of drug resistance for chemotherapy of breast cancer is plasma membrane transporters, including P-glycoprotein (Pgp/ABCB1) and MDR-related proteins (MRPs/ABCC), can actively transport chemotherapy drugs to the extracellular space, ultimately reducing intracellular concentrations [4]. However, the progression of tumor is not controlled by cancer cells exclusively. Tumor cells exposed to low extracellular pH conditions undergo many changes and it is becoming increasingly evident that acidosis plays an important role in drug resistance. The acid-outside pH gradient of solid tumors enables to reduce uptake and efficacy of weak base chemotherapeutics, leading to physiological drug resistance [5]. The acidic environment of tumor cells also reduce killing efficacy of T lymphocyte [6,7]. In order to avoid the potentially lethal consequences of excessive acidification of the cell microenvironment, cancer cells can automatically up-regulate some molecules such as carbonic anhydrase 12 (CA12) and maintain stable pH gradient to promote the survival, proliferation, and invasion of cancer cells [8]. This phenomenon may give rise to escape immune surveillance functions in patients with cancer [9].

CA12 is a transmembrane protein involving in cellular pH regulation of metabolically active cells/tissues by catalyzing a reversible reaction of carbon dioxide hydration and dehydration: H_2_O + CO_2_ ⇄ H + HCO3− [10]. CA12 has been identified as a potential target for therapeutic applications because of its over-expression in human malignant tumors [11]. CA12 can be up-regulated concurrently and interacted with P-glycoprotein in chemotherapy-resistant cells. CA12 silencing decreased the ATPase activity of Pgp by altering the optimal pH at which Pgp operated and promoted chemosensitization to Pgp substrates in MDR cells [12]. Paclitaxel, which is a substrate of P-glycoprotein, is the first-line drug for breast cancer chemotherapy [13]. However, many breast cancer patients have developed chemotherapy failure because of the occurrence of paclitaxel (PTX) resistant cells. Currently, no drugs specifically targeting PTX-resistant breast cancer are available. Therefore, therapies targeting CA12 have recently attracted extensive attentions.

In the present study, we used a popular breast cancer cell line MCF-7 as a model because MCF-7 cell line has positive estrogen receptor (ER) and progesterone receptor (PR) [14], which are excellent targets for chemotherapy. Previous study revealed that CA12 expression was strongly regulated by estrogen and its receptor [15]. Therefore, we hypotheses that CA12 high expression is closely associated with drug-resistance of PTX in breast cancer patients. To address this hypothesis, we performed CA12 silencing in the PTX-resistant breast cancer cell by small interfering RNA transfection (siRNA) and observed the effects of CA12 siRNA on the relevant expressions of the mitochondria signal pathway proteins and proliferation of the PTX-resistant breast cancer cell. This study aimed to explore mechanisms of paclitaxel chemoresistance. These results provided a theory foundation for targeting CA12 therapy.

## Material and methods

### Reagents, kits, and antibodies

A serine protease inhibitor phenylmethylsulfonyl fluoride (PMSF) was purchased from Abcam (Cambridge, MA, USA). Radioimmunoprecipitation assay (RIPA) buffer was purchased from Beyotime (Shanghai, China). Bicinchoninic acid (BCA) protein assay kit and polyvinylidene difluoride (PVDF) membrane were purchased from Bio-RAD (Bio-Rad Laboratories, CA, USA). Sodium dodecyl sulfate–polyacrylamide gel electrophoresis (SDS-PAGE) was purchased from Bioss Antibodies (Beijing, China). Chemiluminescent horseradish peroxidase substrate (cat. no. WBklS0500) was purchased from EMD Millipore (Billerica, MA, USA). 3-(4, 5-dimethylthiazol-2-yl)-2,5-diphenyltetrazolium bromide (MTT, cat. no. M5655), dimethylsulfoxide (DMSO, cat. no. D2650), Triton X-100 (cat. no. H9284), 2 mmol/L sodium orthovanadate (Na3VO4; cat. no. S6508), sodium fluoride (cat. no. S7920), 1 mmol/L edetic acid (cat. no. E9884), PMSF (cat. no. 78,830), aprotinin (cat. no. A11530), and leupeptin (cat. no. L2884) were purchased from Sigma-Aldrich (St. Louis, MO, USA). Dulbecco’s modified Eagle’s medium (DMEM), high-glucose DMEM, fetal bovine serum (FBS), 0.25% trypsin and Opti-MEM®Medium were purchased from Gibco (Thermo Fisher Scientific, Inc., Waltham, MA, USA). Hoechst 33,342 (cat. no. C1025) was purchased from Beyotime Chemicals (Suzhou, China). Rabbit monoclonal CA12 (cat. no. ab195233) and rabbitβ-actin antibodies (cat. no. ab8227) were purchased from Abcam (Cambridge, MA, USA). The proteins of interest were detected using the following primary antibodies: goat polyclonal cleaved caspase-9 (cat. no. sc-22,182; 1:1,000 dilution), mouse monoclonal cleaved caspase-7 (cat. no. sc-56,063; 1:1,000 dilution), rabbit polyclonal Bax (cat. no. sc-493; 1:500 dilution), rabbit polyclonal Bcl-2 (cat. no. sc-492; 1:1,000 dilution), mouse monoclonal Bid (cat. no. sc- 135,847; 1:1,000 dilution), mouse monoclonal Bcl-XL (cat. no. sc-8392; 1:1,000 dilution), rabbit polyclonal PARP (cat. no. sc-7150; 1:1,000 dilution) were purchased from Santa Cruz Biotechnology, Inc. (Santa Cruz, CA, USA). Enhanced chemiluminescence (ECL) reagent was purchased from GE Healthcare (Arlington Heights, IL, USA). CA12-siRNA (cat. no. AM51331), Scramble siRNA (cat. no. AM439083) and Lipofectamine® RNAiMAX Transfection Reagent (cat. no. 13,778,110) were purchased from Thermo Fisher Scientific (USA).

### Cell culture

Human breast MCF-10A cell line was purchased from Shanghai Institute of Biochemistry and Cell Biology, Chinese Academy of Sciences (CAS). The human breast cancer cell (BCC) line MCF-7 was purchased from American Type Culture Collection (ATCC, Manassas, VA, USA). Human PTX-resistant breast cancer MCF-7 TaxR cell line was provided by the Institute of Basic Medical Science, China Academy Medical Sciences (CAMS). All cells were cultured in Dulbecco’s Modified Eagle Medium (DMEM) media containing 10% fetal bovine serum (FBS), 100 µg/mL streptomycin,100 U/mL penicillin, and maintained at 37°C with 5% CO_2_ in a humidified atmosphere.

### Cell transfection

Lipofectamine® RNAiMAX Transfection Reagent was used for siRNA transfection following standard protocol [16]. One day before transfection, MCF-10A, MCF-7, and MCF-7 TaxR cells were seeded into a 6-well plate at a density of 4.0–4.5 × 10^5^ cells per well overnight in high-glucose DMEM media containing 10% FBS without antibiotics. First, the A and B solutions were separately prepared. Solution A: add Lipofectamine® RNAiMAX Transfection Reagent (9 μl) to the Opti-MEM®Medium (150 μl). Solution B: add 3 μl of CA12-siRNA (10 μM) to the Opti-MEM®Medium (150 μl). Then, the diluted A solution with the B solution (1:1) was mixed and placed at room temperature (RT) for 5 minutes. Finally, the solution complex (25 pmol siRNA in each well or formulation) was incubated for 24 hours at 37°C in a 5% CO_2_ atmosphere. At the same time, we also set up the negative control group (Scramble-siRNA), or the blank control group (DMSO treatment) as siRNA culture conditions.

### Western blot analysis

Western blot was performed following previous description [17]. Cytoplasmic proteins of different groups were lysed with lysis buffer (RIPA:PMSF = 100:1) on ice for 30 minutes, and the cell lysate was centrifuged at 13,000 rpm at 4°C for 10 minutes, then the supernatant was collected and stored at −80°C. Protein concentration in the supernatants was determined by BCA Protein Assay Kit. After the proteins were heated at 95°C for 5 minutes to denature, an equal amount of each cell component was loaded into each well of a precast 10% SDS-PAGE gel. After electrophoresis, the proteins were transferred to the PVDF membranes in the transfer buffer. Soak the membrane in blocking buffer (Tris-buffered saline with 0.1% Tween® 20 detergent (TBS-T) buffer and 5% skimmed milk) and incubate at 37°C for 1 hour, followed by overnight incubation with specific primary antibodies at 4°C and then wash four times with TBS-T buffer. Then, the membranes were incubated with the corresponding secondary antibody at room temperature for 1 hour. After that, immunoblotting with chemiluminescent horseradish peroxidase substrate and imaging using imageQuant 350 system (GEHealthcare life Sciences, Piscataway, NJ, USA). Quantify each band by imaging software. The experiment was repeated three times, and the data represents repeated experiments.

As mentioned earlier, the expression of proteins Bax, Bcl-2, Bid, Bcl-xl, cleaved caspase-7, cleaved caspase-9, and cleaved PARP related to the mitochondrial pathway were analyzed using Western blot.

### MTT

Cell proliferation was quantified by standard products. 3-(4, 5-dimethylthiazol-2-yl)-2,5-diphenyltetrazolium bromide (MTT) reduction assay following previous method [18]. Briefly, after digesting by trypsin, MCF-10, MCF-7, and MCF-7 TaxR cells were divided into eight groups, seeded into 96-well plates at a density of 5 × 10^4^/ well, and incubated in DMEM containing 10% heat-inactivated FBS for 24 hours. The cells were treated with the PTX solutions at various concentrations (0 nM, 1 nM, 3 nM, and 5 nM).After 48 hours of incubation, 10 µl of MTT (Sigma,U.S.A.) was added to the cells(4 h) for MTT assay. Remove the supernatant and add 150 µl of stop buffer DMSO to each well. Measure the absorbance at 570 nm with a microplate reader (Thermo, U.S.A.). The calculated results were cell survival rate (%) = [OD 570 nm (drug) /OD 570 nm (control)] × 100%. The half maximal inhibitory concentration (IC50) was calculated from the tumor cell survival curve. Then, each experiment is carried out at least 3 times, and then the average value is calculated. The MCF-7 TaxR negative transfection group and the experimental group were prepared with the same method as the above method, cell suspension was prepared, divided into 5 groups, and added PTX working solutions with final concentrations of 0 nM, 1 nM, 3 nM, and 5 nM, respectively. The remaining steps were as same as the previous method.

### Phase contrast microscope and fluorescence inverted phase contrast microscope

Briefly, according to the measurement result of MTT, 3 nM was selected as the administration concentration of the fluorescence staining experiment of PTX. MCF-10, MCF-7, and MCF-7 TaxR cells were seeded into 6-well plates, respectively, and then Hoechst 33,342 was added for staining. After transfection with DMSO, scramble siRNA, CA12-siRNA plus PTX, the apoptosis of MCF-7 and MCF-7 TaxR was detected by phase contrast microscope (Olympus, Japan) and inverted fluorescence microscope (A350 nm ultraviolet light).

### Flow cytometry

MCF-7 and MCF-7 TaxR cells were seeded into 6-well plates, and then the corresponding siRNA transfection and PTX were processed. According to the manufacturer’s protocol, cells were detected by Annexin V-FITC/PI double staining. As mentioned earlier, use flow cytometry to analyze each sample within 1 hour to determine the rate of apoptosis. Each experiment is carried out at least 3 times, and then the average value is calculated.

### Statistical method

All data were expressed as mean values ± SEM from at least three independent experiments. Statistical significance was determined by one-way analysis of variance with Bonferroni correction and two-way analysis. Experiments were repeated three times, and data are representative of triplicate experiments.

## Result

### CA12 is predominantly over expressed in PTX-resistant breast cancer

**Figure 1.**
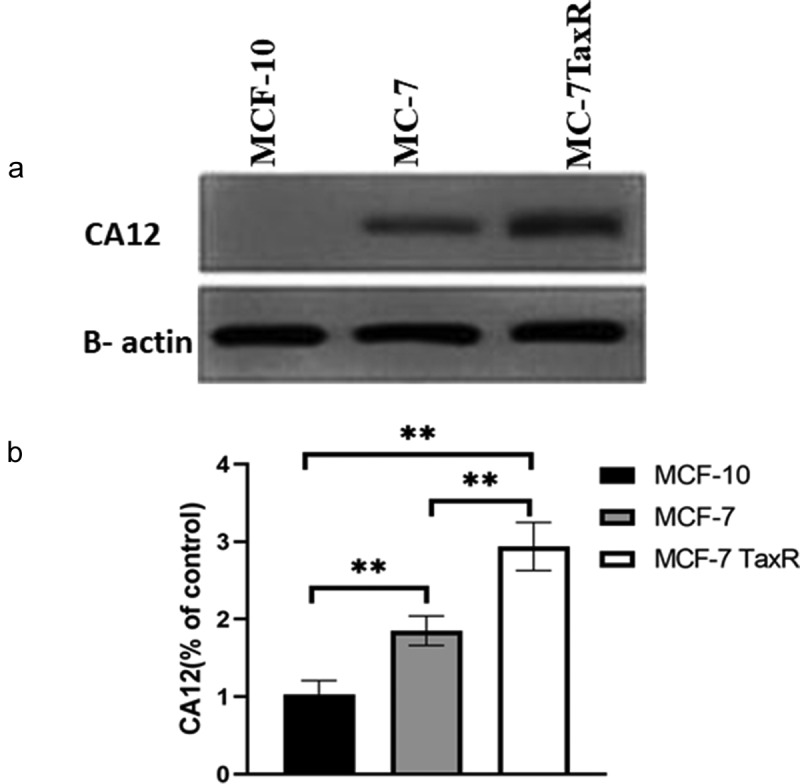
Expression of CA12 protein in different breast cell lines (x ± s, n = 3) by western blot.(a) The expression of CA12 protein in different breast cell lines; (b) The relative expression of CA12 protein in different breast cell lines; **: Comparison between groups, P < 0.01

To investigate whether CA12 protein expression in normal breast cell, breast cancer cells, and PTX resistant breast cancer, we measured CA12 protein levels by western blotting. The results showed that CA12 protein has expressed in MCF-7 and MCF-7 TaxR cell lines, but no expression in MCF-10 cell line (Figure 1(a)). Interestingly, CA12 protein level of MCF-7 TaxR was dramatically higher than that in MCF-7 cell (p < 0.01, Figure 1(b)). Therefore, we hypothesized that CA-12 might contribute to the resistance of breast cancer. To further validate our hypothesis, we performed quantitative real-time polymerase chain reaction, and CA-12 transcription was strongly increased in TaxR resistant cells (data not shown). Taken together, our data indicated that CA-12 was overexpressed in PTX resistant breast tumors.

### Silencing of CA-12 significantly inhibited its expression in TCF-7 and TCF-7 Tax resistant cells

**Figure 2.**
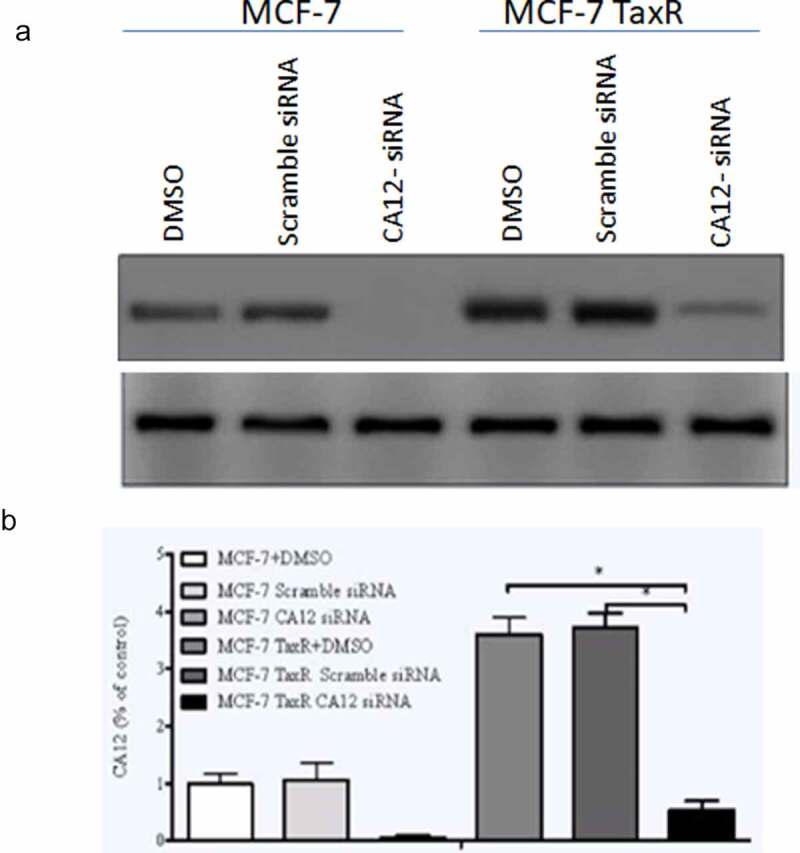
Effects of siRNA transfection on the expression of CA12 protein in MCF-7 and MCF-7 TaxR cells (n = 3). (a)The expression of CA12 protein in MCF-7 and MCF-7 TaxR cells after siRNA transfection; (b) The relative expression of CA12 protein in MCF-7 and MCF-7 TaxR cells after siRNA transfection. *: Compared with the two control groups (blank+negative), P < 0.05

In order to evaluate relationship between CA12 expression and breast cancer cell proliferation, we firstly performed CA12 silencing in MCF-7 and MCF-7 Tax R cells and observed whether CA12-siRNA inhibits the expression of CA12 after transfection with different reagents in the breast cancer cell lines. The present study compared the protein levels of CA12 in three groups by western blot. As shown in Figure 2, the relative expression of CA12 protein in the blank control group (DMSO), negative control group (Scramble siRNA), and experimental group (CA12-siRNA) of MCF-7 and MCF-7 TaxR cells (Figure 2(a)). We found that CA12-siRNA can completely abolish CA12 gene expression in MCF-7 cells and inhibit the most CA12 gene expression in MCF-7 TaxR (Figure 2(b)). As predicted, our results revealed that CA12-siRNA can significantly block CA12 expression in MCF-7 and MCF-7 TaxR cells.

### CA12 silencing was significantly enhanced the sensitivity to PTX in MCF-7 TaxR

**Figure 3.**
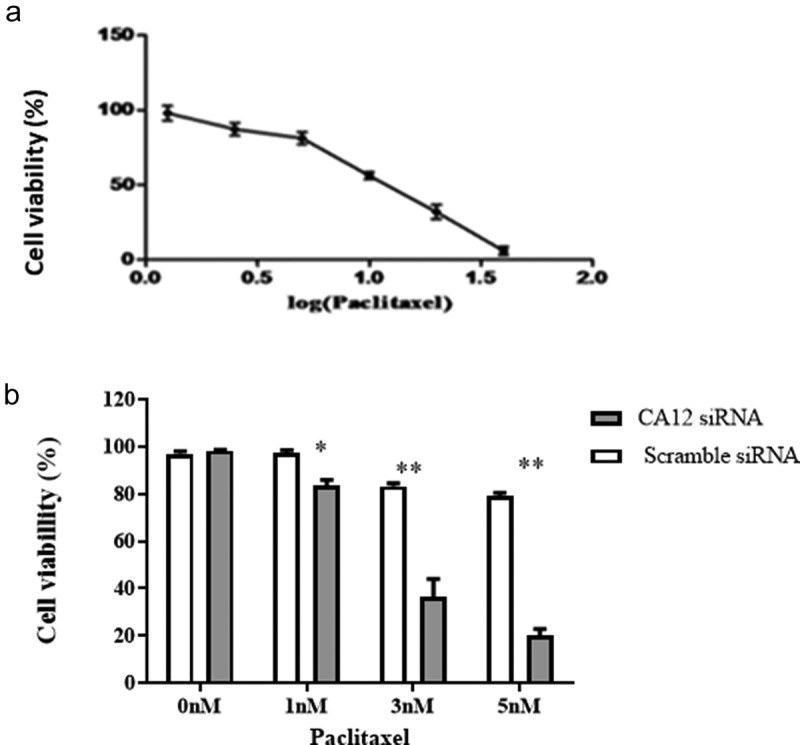
After CA12-siRNA transfection the survival rates of MCF-7 TaxR cells.(a) Survival curve of MCF-7 TaxR cells treat with paclitaxel (x ± s, n = 3); (b) Paclitaxel sensitivity of MCF-7 TaxR after CA12-siRNA transfection (x ± s, n = 3). Compared with the negative control group (Scramble siRNA), *P < 0.05;**P < 0.01

To further evaluate the effects of CA12 siRNA on proliferation of MCF-7 TaxR cells with PTX co-culture. We performed the MTT assay to measure the proliferation activity of MCF-7 TaxR cell after a serial of concentrations of PTX treatments and calculate the cell survival rate. Then, the survival curve of the MCF-7TaxR cells was obtained (Figure 3(a)). The results showed that the survival rate of MCF-7 TaxR cells gradually decreased with PTX dose increase in a concentration-dependent manner. The half inhibitory concentration (IC50) of PTX in MCF-7 TaxR cells was 10.43 ± 1.56 nM, and the determination coefficient was R2 = 0.9887.

In the next step, survival rate of MCF-7 TaxR cell in either PTX combined with the corresponding siRNAs or the negative control group for 48 hours were compared (Figure 3(b)). The results showed that in the absence of PTX, there was no significant difference in cell survival between the negative control group (Scramble siRNA) and the experimental group (CA12-siRNA) (P > 0.05).After the corresponding siRNA and PTX combined treatment, the survival rate of the two groups of cells decreased with the increase of PTX concentration. Interestingly, the survival rate of cells in the experimental intra-groups was significantly lower than that in the negative control group at the same concentration, and the difference was statistically significant (P < 0.05, Figure 3(b)). It was shown that the sensitivity of MCF-7 TaxR cells to PTX was dramatically increased in a PTX dose dependent manner after CA12-siRNA transfection (*P*< 0.01).

### CA12-siRNA transfection combined with PTX induces apoptosis of MCF-7 TaxR cells

**Figure 4.**
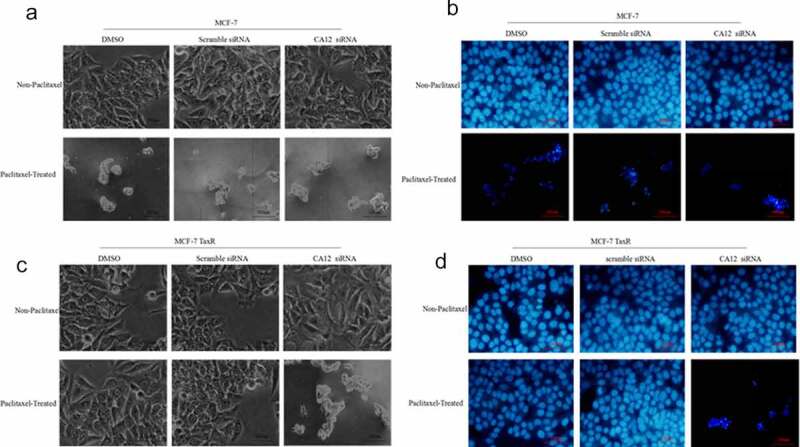
CA12-siRNA transfection combined with paclitaxel induces apoptosis of MCF-7 TaxR cells. (a) Morphological observation of MCF-7 cells after siRNA transfection; (b) Hoechst 33,342 fluorescence staining observation of MCF-7 cells after siRNA transfection;(c) Morphological observation of MCF-7 TaxR cells after siRNA transfection; (d) Hoechst 33,342 fluorescence staining observation of MCF-7 TaxR cells after siRNA. Scale bar,100 μM

To validate mechanism of CA12 silencing in MCF-7 TaxR, we monitored MCF-7 and MCF-7 TaxR cell morphology changes with and without PTX administration. MCF-7 and MCF-7 TaxR cells were treated with the DMSO, scramble siRNA, and CA12-siRNA. The cell morphologies in different conditions were recorded under inverted phase contrast microscope and fluorescent microscope (after Hoechst 33,342 staining) (Figure 4).

Without PTX administration, DMSO and scramble siRNA treated MCF-7 cell line grew well. Size of CA12-siRNA treated MCF-7 cells just became smaller, but no significant differences between CA12-siRNA and control treated cells. However, with siRNA combined PTX treatment for 48 hours, a large amount of MCF-7 cells in all three groups fell off, which a few remaining parts decreased, poor refractive index, shrinkage apoptotic cells (Figure 4(a)). Similar phenomena were observed under a fluorescence microscope (Figure 4(b)).

In contrast, MCF-7 TaxR cells were treated with PTX plus CA12-siRNA, DMSO or scramble siRNA .The MCF-7 TaxR cell line grew well without obvious apoptosis with PTX plus DMSO or scramble siRNA treatment. However, in CA12 siRNA treated group, MCF-7 TaxR cells died in a large area, leaving only a few apoptotic cells with atrophy of nucleus and cytoplasm (Figure 4(c)). Significant differences were observed with and without PTX treatment. Under the same circumstances, similar phenomena were identified under a fluorescence microscope (Figure 4(d)). This result suggested that CA12 silencing can promote the apoptosis of MCF-7 TaxR, and then reverse the PTX resistance.

### Flow cytometry to detect apoptosis rate

**Figure 5.**
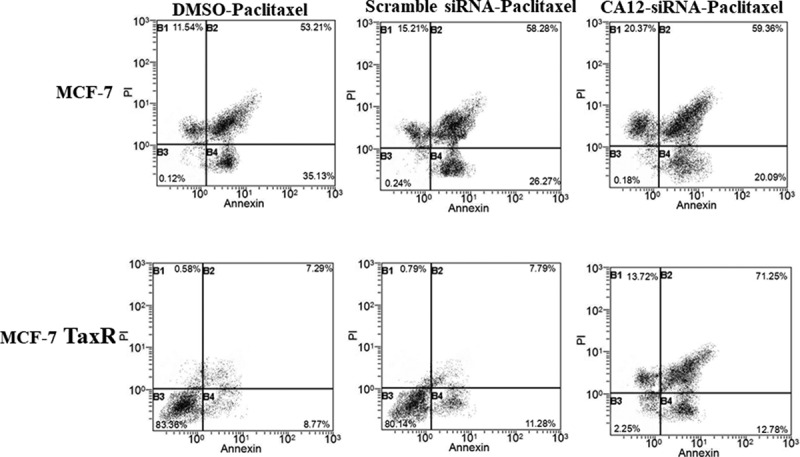
Flow cytometry to detect apoptosis rate. Apoptosis in MCF-7 and MCF-7 TaxR cells after combination siRNA transfection with paclitaxel culture. Compared with the blank control group, *P < 0.05;**P < 0.01. Compared with the negative control group, ^#^P < 0.05; ^##^P < 0.01

To confirm that CA12 silencing enhanced apoptosis of MCF-7 TaxR cells, we used FACS to quantity apoptotic cell number. The same grouping and treatment as above, MCF-7 cells were transfected with CA12 siRNA at 3 nM paclitaxel for 48 hours. From previous experiment, data showed that CA12-siRNA had dramatic inhibitory effects on MCF-7 Tax R cell at 3 nM paclitaxel administration. Annexin V-FITC/ propidium iodide (PI) double staining was used to detect apoptotic cells It was demonstrated that the DMSO, scramble siRNA, and the CA12-siRNA treated MCF-7 cells had 53.21%, 58.28%, and 59.36% apoptotic cells, respectively. For MCF-7 TaxR cells, the percentage of apoptotic cells in the DMSO, scramble siRNA, and CA12-siRNA groups were 7.29%, 7.79%, and 71.25%, respectively (Figure 5).This data confirmed that CA12 silencing can promote apoptosis of MCF-7 TaxR cells.

### Mitochondrial apoptosis pathway activation

**Figure 6.**
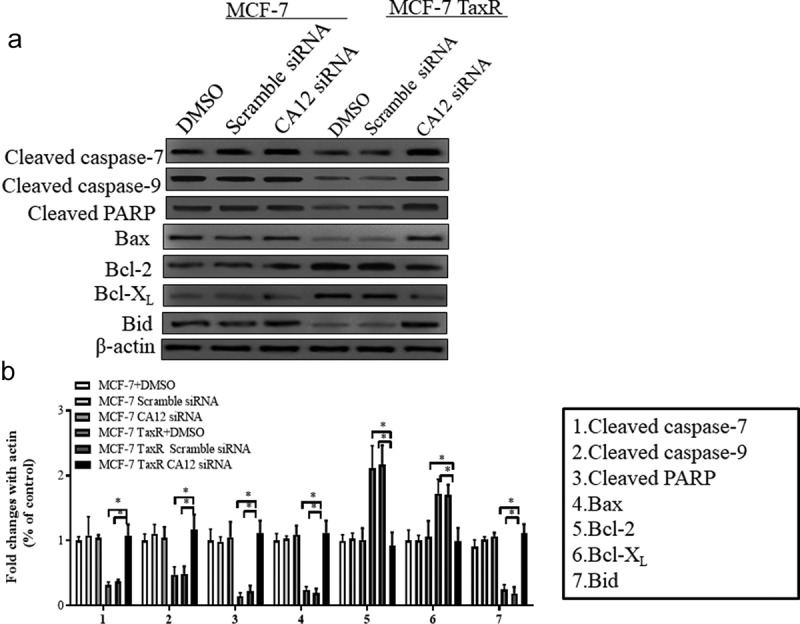
Effects of siRNA transfection and paclitaxel culture on mitochondrial apoptotic signaling pathway in MCF-7 and MCF-7 TaxR cells. (a) Expression of mitochondrial apoptosis-related protein in MCF-7 and MCF-7 TaxR cells;(b) The relative expression of mitochondrial apoptotic pathway-related proteins in MCF-7 and MCF-7 TaxR cell. *: Compared with the experimental group (CA12-siRNA), P < 0.05

To further investigate mechanism of CA12 silencing in PTX chemotherapy, we speculated that mitochondrial apoptosis signaling pathway, which is also known as the intrinsic pathway, are involved in anti-tumor effects of PTX. As we know, tumor cells are easy to undergo intrinsic pathway because of its sensitivity to anti-tumor drugs [19]. With initiative activation of intrinsic pathway, the proportion between proapoptotic proteins like BaX, BID, BAD and anti-apoptotic proteins such as Bcl-2 and Bcl-XI on the outer-membrane of mitochondrion determines the fate of downstream signal molecules [20]. Therefore, we explored the relevant protein expressions of mitochondrial apoptosis signaling pathways in the CA12 silencing of MCF-7 and MCF-7 TaxR cells. As shown in Figure 6, the change trend of the protein expression in each group of MCF-7 cells is similar, and there is no statistical difference among the groups by CA12 silencing (Figure 6(a)). However, compared with the blank/negative control, the expression of anti-apoptotic proteins Bcl-2 and Bcl-XL in MCF-7 TaxR cells in the experimental group was significantly down-regulated, and expression of pro-apoptotic proteins (Bax, Bid, Cleaved caspase-7, Cleaved caspase-9, Cleaved PARP) were up-regulated in the presence of CA12 siRNA (Figure 6(b)). This result demonstrated that CA12 silencing in PTX treated MCF-7 TaxR cells reversed the sensitivity to PTX because CA12 silencing promoted the apoptosis of MCF-7 TaxR cell by up-regulated pro-apoptotic protein and down-regulated anti-apoptotic protein expressions.

## Discussion

Breast cancer is one of the most frequent malignancies and the leading cause of mortality from cancer in women [21]. With the development of various treatments, the prognosis of breast cancer patients has improved in the past few decades [22]. However, required drug resistance is still a major problem of cancer in improving the outcome of breast cancer patients. Unfortunately, paclitaxel (PTX) as the first-line of chemotherapy for breast cancer patients was reported to develop drug-resistance in 90% of breast cancer patients associated with toxic side effects and poor solubility [23,24]. The mechanisms of PTX resistance have not been fully understood, which are associated with poor response and metastases, and the main cause of death in breast cancer patients [25,26]. Some evidence showed that PTX resistance may be relevant to over-expression of P-glycoprotein, efflux of chemotherapeutic drugs, changes in tubulin dynamics, and so on [27]. Therefore, investigating exact molecular mechanism of PTX resistance and developing the strategy improving its therapeutic efficiency are crucial to breast cancer cure.

CA12, a single transmembrane protease containing zinc metal, can catalyze a reversible reaction of carbon dioxide hydration and dehydration in tumor tissues, thereby regulating the pH of the extracellular microenvironment [28]. Previous experiments have shown that CA12 expression in breast cancer cells was significantly up-regulated and was strongly associated with low tumor grade, estrogen receptor (ER) positive, and epithelial growth factor receptor (EGFR) negative status [29,30]. CA12 can regulate the pH value of tumor microenvironment and the expression of P-glycoprotein, and then is involved in the processes of tumor cell proliferation, invasion, and drug resistance [12,31,32]. CA12 silencing decreased the ATPase activity of Pgp by altering the optimal pH value of Pgp and promoted chemosensitization of MDR cells to Pgp substrates [12]. In order to confirm these findings, this study used western blot to detect the expression of CA12 in MCF-10, MCF-7, and MCF-7TaxR cells. The results showed that there were high expressions of CA12 in MCF-7 and MCF-7 TaxR but undetectable in MCF-10 cell. These data indicated that the overexpression of CA12 may be related to breast cancer and PTX resistance.

siRNA is a 19–22 bp RNA duplex, which sequence structure has homology with target mRNA sequence. By integrating its guide strand into the RNA-induced silencing complex (RISC), it can effectively degrade homologous mRNA, thereby inhibiting the expression of the corresponding gene [33]. Contrasted with conventional targeted therapeutics (monoclonal antibodies and small-molecule inhibitors), siRNA has higher intracellular delivery efficiency and more target proteins [34]. Several previous studies have described the therapeutic potential of using siRNA to knock down the corresponding protein in breast cancer [35–38]. Therefore, in the present experiments, we used artificially synthesized CA12-siRNA (experimental group) and scramble siRNA (negative control group) to transfect MCF-7 and MCF-7 TaxR cells, respectively. Western analysis showed that CA12-siRNA significantly reduced the expression of CA12 at the protein level in MCF-7 and MCF-7 TaxR cells. We also noticed that CA12 siRNA can completely abolish CA12 protein expression in MCF-7, but not in MCF-7 TaxR cells, which this may be because CA12 protein level in MCF-7 TaxR cells was significantly higher than that in MCF-7 cells. With and without PTX administration, MTT was used to detect and compare the cell survival rate of MCF-7 TaxR cell line. The results showed that there were no significant changes in survival and apoptosis after transfection with siRNA alone. This indicates that simply inhibiting the expression of CA12 has a limited effect on inducing apoptosis. After the CA12-siRNA transfected cells and PTX were co-cultured for 48 hours, the amount of apoptosis were significantly increased in the experimental group compared with the two control groups (the blank and negative groups),indicating that the combination of siRNA and PTX treatment enhance the chemosensitivity of drug-resistance breast cancer cells. The same results were observed with an inverted phase contrast microscope and a fluorescent inverted phase contrast microscope (following Hoechst 33,342 staining).

Among the various forms of endogenous apoptosis, the mitochondrial apoptotic pathway is the main mechanism utilized by chemotherapy [39], which can be regulated by apoptotic proteins. Apoptotic proteins are divided into two categories: pro-apoptotic proteins and anti-apoptotic proteins. Pro-apoptotic proteins include Bcl-2, Bcl-xl, etc. Anti-apoptotic proteins include Bax, Bak, Bid, etc. [40–42]. When cancer cells are stimulated by cytotoxic drugs, ultraviolet rays or other stimulus, endogenous cell apoptosis is activated [43], and the conformation of the anti-apoptotic protein changes, prompting cells to release the mitochondrial interstitial protein cytochrome C (Cytc) [41,44], which activates downstream effector molecules caspase-7, caspase-3 and PARP activation proteins Cleaved caspase-7, Cleaved caspase-9, inhibit DNA repair, and ultimately lead to cell apoptosis [45].

Here, to explore the apoptosis pathway induced by the combination of CA12-siRNA and PTX, we detected Bid, Bax (pro-apoptotic protein), Bcl-xl, Bcl-2 (anti-apoptotic protein) in each group of cells. And the expression of activated caspase-7 (Cleaved caspase-7), activated caspase-9 (Cleaved caspase-9), activated PARP (Cleaved PARP) was also determined by western blot. Previous studies [27] have consistently shown that the cytotoxicity of PTX lies in its ability to initiate the mitochondrial apoptotic pathways, such as Bcl-2 hyperphosphorylation and mitochondrial calcium efflux or influx resulting in programmed cell death. It was documented that high-dose PTX (above 12 nM) can reduce the proliferation of breast cancer MCF-7 cells, depending on the mitochondrial apoptotic pathway, while low-dose PTX does not involve this pathway [46,47]. Therefore, we choose 3 nM PTX to exclude the effects of high concentrations of PTX on mitochondrial apoptosis pathway-related proteins. Western analysis showed that after CA12-siRNA and PTX (3 nM) treatment, the anti-apoptotic proteins Bcl-xl and Bcl-2 of MCF-7 TaxR cell line were significantly reduced, while the pro-apoptotic proteins Bid, Bax and related downstream effector molecules were increased significantly. However, there was no statistical difference in the expression of apoptosis-related proteins in MCF-7 cells treated with the same treatment. Here, we focused on mitochondrial pathway of apoptosis because chemotherapy drugs generally trigger this pathway. CA12-siRNA may also be involved in other pathways of apoptosis except mitochondrial pathway to be explored in the future. Thus, the combination of CA12-siRNA and PTX may induce drug-resistant cells by activating the mitochondrial apoptosis pathway apoptosis, thereby reversing the resistance of breast cancer cells to PTX.

## Conclusion

PTX is the first-line drug for breast cancer chemotherapy. PTX resistance of breast cancer cell is the main reason for treatment failure in most breast cancer patients. Here, we cultivated a PTX resistant MCF7-TaxR breast cancer cell line with CA12 siRNA and found that CA12 silencing can enhance the chemosensitivity of MCF7-TaxR to PTX by activating the mitochondrial apoptosis pathway.

## Data Availability

Data are available from the corresponding author on request.

## Highlights

- The CA12 protein has high expression in breast cancer cell line MCF-7 and MCF-7 TaxR.
- The CA12 gene silencing was dramatically promoted the sensitivity of paclitaxel in paclitaxel-resistant cell line MCF-7 TaxR.
- The CA12 silencing in MCF7 TaxR cells activated the mitochondrial apoptosis pathway and promoted MCF-7 TaxR apoptosis.
- Targeted CA12 may provide a new strategy for the treatment of paclitaxel-resistant breast cancer.
